# Study of the relationship between regional cerebral saturation and pCO_2_ changes during mechanical ventilation to evaluate modifications in cerebral perfusion in a newborn piglet model

**DOI:** 10.1590/1414-431X2022e11543

**Published:** 2022-02-28

**Authors:** F. Silvera, T. Gagliardi, P. Vollono, C. Fernández, A. García-Bayce, A. Berardi, M. Badía, B. Beltrán, T. Cabral, P. Abella, L. Farías, L. Vaamonde, M. Martell, F. Blasina

**Affiliations:** 1 Department of Neonatology, Hospital de Clínicas Dr. Manuel Quintela, Faculty of Medicine, Republic University, Montevideo, Uruguay; 2 Department of Neonatology, Centro Hospitalario Pereira Rossell, Administración de los Servicios de Salud del Estado, and Faculty of Medicine, Republic University, Montevideo, Uruguay; 3 Division of Pediatric Imagenology, Centro Hospitalario Pereira Rossell, Administración de los Servicios de Salud del Estado, and Faculty of Medicine, Republic University, Montevideo, Uruguay

**Keywords:** NIRS, Carbon dioxide pressure, Newborn, Autoregulation, Cerebral blood flow

## Abstract

Near-infrared spectroscopy (NIRS) could be a useful continuous, non-invasive technique for monitoring the effect of partial pressure of carbon dioxide (PaCO_2_) fluctuations in the cerebral circulation during ventilation. The aim of this study was to examine the efficacy of NIRS to detect acute changes in cerebral blood flow following PaCO_2_ fluctuations after confirming the autoregulation physiology in piglets. Fourteen piglets (<72 h of life) were studied. Mean arterial blood pressure, oxygen saturation, pH, glycemia, hemoglobin, electrolytes, and temperature were monitored. Eight animals were used to evaluate brain autoregulation, assessing superior cava vein Doppler as a proxy of cerebral blood flow changing mean arterial blood pressure. Another 6 animals were used to assess hypercapnia generated by decreasing ventilatory settings and complementary CO_2_ through the ventilator circuit and hypocapnia due to increasing ventilatory settings. Cerebral blood flow was determined by jugular vein blood flow by Doppler and continuously monitored with NIRS. A decrease in PaCO_2_ was observed after hyperventilation (47.6±2.4 to 29.0±4.9 mmHg). An increase in PaCO_2_ was observed after hypoventilation (48.5±5.5 to 90.4±25.1 mmHg). A decrease in cerebral blood flow after hyperventilation (21.8±10.4 to 15.1±11.0 mL/min) and an increase after hypoventilation (23.4±8.4 to 38.3±10.5 mL/min) were detected by Doppler ultrasound. A significant correlation was found between cerebral oxygenation and Doppler-derived parameters of blood flow and PaCO_2_. Although cerebral NIRS monitoring is mainly used to detect changes in regional brain oxygenation, modifications in cerebral blood flow following experimental PaCO_2_ changes were detected in newborn piglets when no other important variables were modified.

## Introduction

In the first days of life, changes in oxygenation or fluctuations in the cerebral blood flow (CBF) play a major role in brain injury (periventricular leukomalacia, intraventricular or parenchymal hemorrhage) and in long-term neurodevelopment impairment (1).

The primary mechanisms regulating CBF are PaCO_2_, mean arterial blood pressure (MA BP), cerebral metabolism, and the autonomous nervous system. Among these, cerebral perfusion is extremely sensitive to changes in PaCO_2_ (3-6% increase and/or 1-3% decrease in flow per mmHg change in CO_2_ above or below normocapnia levels, respectively) (2). The entire vascular tree is vasoactive. This increased vascular sensitivity to CO_2_ manifests throughout the cerebrovascular bed, from the large intracranial arteries to the smallest pial arterioles and cerebral parenchymal vessels. While the network of small arterioles in the pia mater modulates the regional blood flow, the large vessels serve as the “front line” to maintain stable cerebral perfusion (2). In the hypercapnic range, the sensitivity to changes in CO_2_ is similar between brain regions, but in hypocapnia, differential vascular sensitivity is observed in the regions irrigated by the posterior cerebral arteries and, therefore, CO_2_ reactivity is higher in grey matter than in white matter (2).

The cerebral vasculature is also dependent on arterial blood PaO_2_, but only below a certain threshold (i.e., 50 mmHg - 80% arterial saturation). Moreover, the CBF response to hypoxia is also dependent on PaCO_2_ (hypercapnia increases and hypocapnia reduces the cerebral sensitivity to hypoxia) (3).

The cerebral vasculature of the mature brain has the ability to maintain stable cerebral perfusion within a certain range, despite changes in systemic blood pressure. This mechanism, known as cerebral autoregulation (CA), can be easily altered in newborns, especially preterm newborns, which is particularly important in states of low systemic blood pressure (4).

CA has been evaluated with transcranial Doppler (TCD) ultrasound by bedside measurement of cerebral blood flow velocity, which serves as an estimator of CBF, and by monitoring the regional cerebral tissue oxygen saturation (rSaO_2_) by using near-infrared spectroscopy (NIRS).

The use of NIRS to study changes in hemodynamics and cerebral oxygenation of the human newborn was first described in 1985 by Brazy et al. (5) and Naulaers et al. (6). This technology has been used for several years providing continuous real time noninvasive monitoring to obtain relevant information about tissue oxygenation (which is modified by the blood flow, hemoglobin concentration, and metabolic tissue rate) in different pathologies (7). Assuming that cerebral metabolism is relatively constant, as well as O_2_ consumption, and that the volume of venous and capillary blood is stable, changes in cerebral rSaO_2_ should reflect regional CBF (5). The fractional tissue oxygen extraction (FTOE) evidences the regional balance between oxygen supply and consumption as another important index obtained from NIRS monitoring to make bedside decisions to assist newborn patients in critical condition (8). The cerebral vasoreactivity to PaCO_2_ has been studied using NIRS, evaluating both volume and CBF (9,10). It has been proven that the use of NIRS in preterm infants allows to estimate the ratio of cerebral blood volume reactive to CO_2_ in a wide range of PaCO_2_ values. Additionally, alterations in CA have been identified in this group of patients using cerebral rSaO_2_, showing consistency between changes in MA BP and the NIRS recordings of patients presenting intraventricular hemorrhage and periventricular leukomalacia (11).

Evidence shows that extremely low levels of PaCO_2_ are associated with cerebral vasoconstriction and periventricular leukomalacia, leading to cerebral palsy (11). This is particularly relevant in patients under mechanical ventilatory assistance, where hyperventilation can lead to hypocapnia. Low rSaO_2_ levels can be good indicators of the need to optimize ventilatory settings (12). Hypercapnia, on the other hand, is associated with cerebral vasodilation and intracranial hemorrhage (13). Currently, strategies like permissive hypercapnia (PaCO_2_ 45-55 mmHg) are common during mechanical ventilation and have shown to reduce ventilator-induced lung injury. However, such beneficial effect has not been observed in the brain, and studies have shown contradictive results on the matter (14,15). The influence of the different ventilation modes on neonatal CBF has been proven (16). It is known that high-frequency ventilation (HFV) can result in rapid changes in PaCO_2_ with hypocapnia as a result (17). Usually, respiratory monitoring is conducted by pulse oximetry and blood gas tests. Non-invasive methods, such as measurement of end-tidal CO_2_ levels and transcutaneous measurement of CO_2_, have an important role in the monitoring of the trends of PaCO_2_ levels. However, these methods are not able to identify physiological alterations secondary to significant changes in PaCO_2_ (18).

The evaluation of changes in PaCO_2_ and CBF using a non-invasive technique such as NIRS in ventilated piglets has a translational value in the neonatal clinical practice, even in states of normoxia.

The main objective of this work was to evaluate the efficacy of NIRS in detecting modifications in CBF secondary to acute changes in PaCO_2_ in a newborn piglet model under mechanical ventilatory assistance after confirming the normal cerebral autoregulation in this piglet species. As a complementary objective, the authors aimed at explore the use of NIRS as a tool to improve the management of the patient in the intensive care unit, deepening the knowledge of the factors that affect the value of rSaO_2_.

## Material and Methods

Newborn piglets (<72 h of life) (*Susscrofa domestica)* were acquired from a local farm under standardized conditions and adequate for experimental research (Granja La Familia, Uruguay).

### Protocol approval

The study was conducted according to the ethical principles that regulate animal research issued by the Helsinki Declaration, the American Physiological Society, and the Uruguayan Institutional Animal Care and Use Committee (CHEA) of the Republic University, Authorization No. 070153-000645-15 (http//www.expe.edu.uy/expe/srchsiteResoluciones.nsf/multidataBaseSearch_Resol), which approved the experimental protocol.

### Piglet model

Animals were anesthetized with ketamine 30 mg/kg *im*, and a mixture of ketamine 3 mg·kg^-1^·h^-1^, midazolam 0.3 mg·kg^-1^·h^-1^ *iv*, and fentanyl 10 μg·kg^-1^·h^-1^ *iv* was added for anesthetic maintenance. Throughout the experiment, piglets received parenteral hydration with 5% glucose at a rate of 70 mL·kg^-1^·day^-1^ to account for the basal intake of fluids and glucose.

Airway access was obtained by tracheostomy with the animal connected to a mechanical ventilator (Babylog^®^ 8000plus, Dräguer, Germany). The initial ventilatory settings were as follows: respiratory rate (RR) of 20 breaths per minute (BPM), positive end-expiratory pressure (PEEP) of 4 cm H_2_O, maximal inspiratory pressure (MIP) to obtain an exhaled tidal volume of 4-6 mL/kg, inspiratory time consistent with the time constant (according to the flow-time curve), and fraction of inspired oxygen (FiO_2_) needed to saturate 90-95%. The gasometric objective of these ventilatory settings were as follows: pH in the range of 7.25-7.35, PaCO_2_ in the range of 35-45 mmHg, PaO_2_ in the range of 50-80 mmHg, and lactic acid <3 mM.

Femoral artery (continuous invasive monitoring of arterial pressure) and femoral venous (perfusion of basal hydration and drugs) catheterism was performed. A single dose of cefradine at 50 mg/kg *iv* and gentamicin at 4.5 mg/kg *iv* was administered in a prophylactic manner. Temperature was maintained between 38.5°C and 39.5°C using a thermal crib and a heater (ThermaCare Heater, Gaymar Industries, USA) with a plastic cover covering the whole animal.

**Figure 1:**
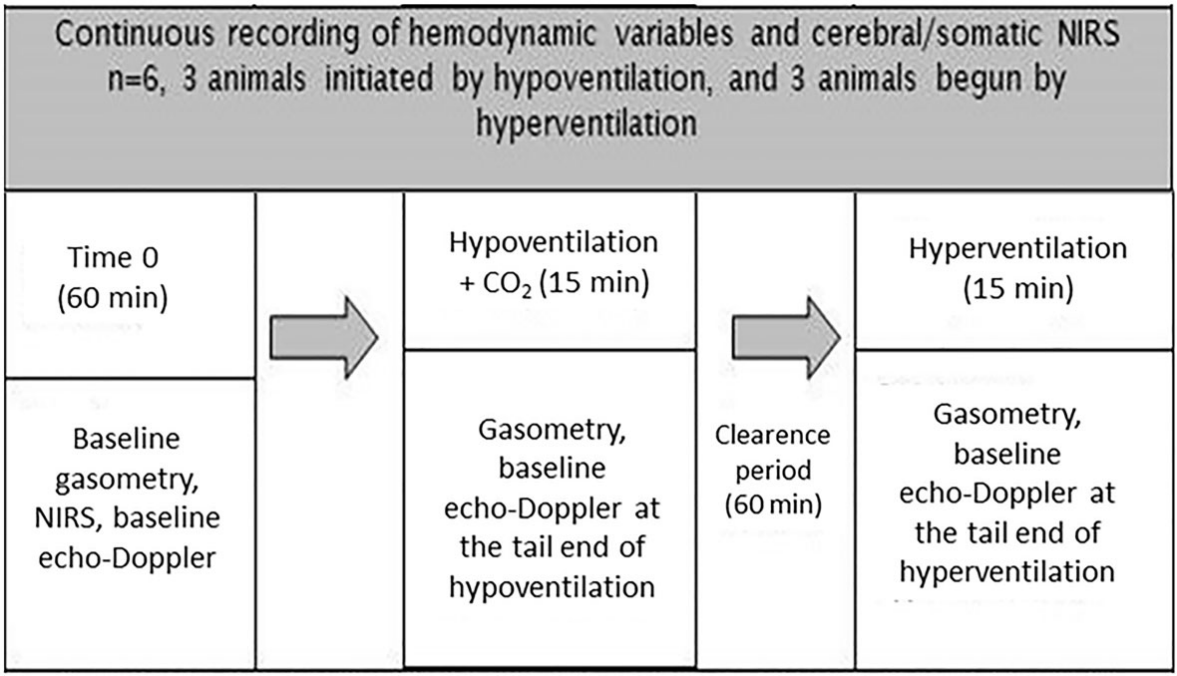
Flowchart showing continuous recording of hemodynamic variables and cerebral/somatic near-infrared spectroscopy (NIRS).

The following parameters were continuously monitored (Figure 1): i) continuous monitoring of oxygen saturation by pulse oximetry and esophageal core temperature (enGuard CM4 monitor, Masimo SET, Ohmeda Medical, USA); ii) electrocardiography with electrodes placed in the upper limbs and the lower left limb (D2 derivative), heart rate (HR), and systolic arterial pressure (SAP) (enGuard CM4 monitor, Masimo SET, Ohmeda Medical); iii) cerebral and splanchnic rSaO_2_ with NIRS by *in vivo* Optical Spectroscopy System [Covidien, Ireland (formerly Sommanetics, USA)] INVOS™ 5100C Cerebral/Somatic Oximeter, with OxyAlert™ (Covidien, Ireland (formerly Sommanetics, USA) NIRS sensors (Cerebral/Somatic Infant-Neonatal Sensors). The somatic sensor was placed in the abdominal region of the animal and the cerebral sensor was located diagonally on the head, covering the fronto-parietal region, avoiding the sagittal sinus's flow.

### Intermittent Doppler ultrasound monitoring

All subjects underwent a color Doppler ultrasound evaluation using a Terason uSMART 3300 device (Teratech Corporation, USA) with a linear array of 12-5 MHz transducer, conducted by the same operator, who was an expert imagenologist with 20 years of experience in vascular Doppler ultrasound in adults and children.

Volumetric blood flow measurement in the internal jugular vein was determined by the device's software using the formula: Volume flow = Cross-sectional area × Time-averaged velocity (TAV) (19,20).

The measurement was made at mid-cervical level and done cautiously to prevent applying pressure on the vessel, in order to minimize the alteration of its diameter. The internal jugular vein (IJV) has an approximately elliptical cross-section, but ultrasound devices assume a circular section, with the cross-sectional area (CSA) calculated as CSA = Diameter^2^ × 0.785. In order to minimize error, measurements of the maximum and minimum diameters were performed in the transverse plane, and the average diameter was used for the calculation of the CSA.

The Doppler spectrum measurements used for the calculation of TAV were obtained in the longitudinal plane. Care was taken to make the Doppler measurement at the same place where the diameter had been measured. Doppler angle of insonation was kept between 30 and 60 degrees. The sample volume was positioned at the center of the vessel, with an amplitude allowing sampling of 50-70% of the lumen.

TAV was automatically calculated by the device, estimated from the intensity-weighted mean frequency (TAMEAN).

Although there is no consistency in the different methods of flow rate color Doppler assessment (21), in our model, we considered this fact of relative importance, since we focused on the relative changes after different interventions rather than on the absolute values of calculated volume flow.

Measurement of CBF by direct vascular Doppler ultrasound was performed on the IJV at baseline and at the end of each intervention (hyperventilation and hypoventilation). It is worth mentioning that TCD ultrasound is the most frequently used method for quantifying CBF (18). Due to the lack of equipment, TCD ultrasound was not used in this study. Thus, this study sets a precedent for future research on estimating CBF by measuring IJV Doppler velocity as a suitable proxy for cerebral volume flow variations.

Blood gases control was calculated at baseline and at the end of each intervention. The fractional tissue oxygenation extraction (FTOE) was calculated with the formula: (Arterial saturation of O_2_ - Cerebral rSaO_2_) / Arterial saturation of O_2_.

### Induced hemodynamic changes/CA

A group of 8 piglets was used to study normal autoregulation. After initial stabilization, the group was divided as follows: 1) 4 animals were prepared as previously described, and after baseline conditions were met, increasing doses of norepinephrine (0.05 to 2 μg·kg^-1^·min^-1^) were administered every 30 min, and 2) 4 piglets in baseline conditions and with confirmed hemodynamic stability were subjected to a period of systemic hypotension caused by increasing doses of sodium nitroprusside (6 to 30 μg·kg^-1^·min^-1^) administered every 30 min.

Brain and somatic NIRS, cava vein blood flow velocity, and left ventricular ejection fraction (LVEF) were measured 15 min after each norepinephrine or sodium nitroprusside dose change. After this intervention, it is not possible to stabilize the animal to perform the other measurements, and for this reason, all animals were euthanized with pentobarbital and potassium, following the approved protocol.

### NIRS Doppler measurements by hypercapnia/hypocapnia intervention

The model included two experimental procedures in the same group of animals (n=6): 1) Hypercapnia: in continuous mandatory ventilation (CMV), delta P was decreased by 9 cm H_2_O (final PIM 6 cm H_2_O) and RR was decreased by 10 BPM (final RR 10 BPM), and supplementary administration of CO_2_ was added to the ventilator gas mixture. FiO_2_ was adjusted to achieve a saturation of 90-95%. Duration of the intervention was 15 min. 2) Hypocapnia: in CMV, delta P was increased by 20 cm H_2_O (PIM 25 cmH_2_O) and RR by 15 BPM (final RR 35 BPM). FiO_2_ was adjusted in order to achieve a saturation of 90-95%. Duration of the intervention was 15 min.

**Figure 2:**
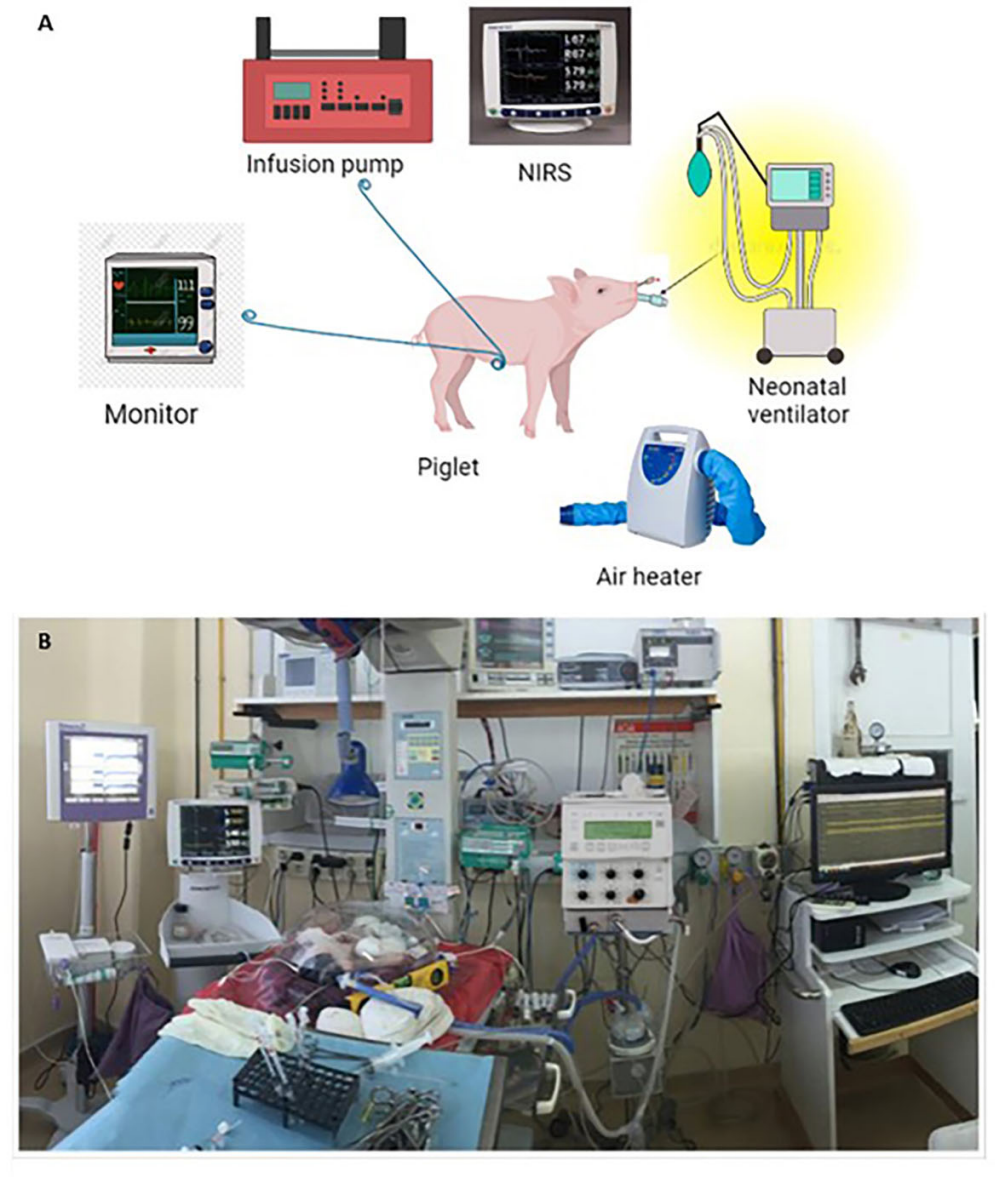
Experimental set-up. **A**, Main equipment used in the experiments; **B**, Photograph of the experimental laboratory. NIRS: near-infrared spectroscopy.

After randomization, hypercapnia/hypocapnia was applied as shown in Figure 2. After the experimental intervention was completed, the animals were euthanized. This experimental protocol was designed with the intention of controlling the variables that have impact on the CBF while maintaining the stability of these parameters during the experimental period. However, the cardiac function can be altered when using increased ventilatory settings to achieve hyperventilation (and hypocapnia). By increasing intrathoracic pressure, such ventilator changes may decrease the systemic venous return and, consequently, decrease the cardiac output.

### Statistical analysis

Statistical analysis was performed by Graph Pad Prism 6 (USA). Student's *t*-test was used to compare results between hyper- or hypoventilation and baseline conditions of CFB, PaCO_2_, and NIRS.

Pearson correlation was used to evaluate PaCO_2_ *vs* NIRS and linear regression was performed to assess the strength of association between CBF and PaCO_2_ or FTOE and PaCO_2_. All data are reported as means±SD. A P value <0.05 was considered statistically significant.

## Results

### Induced hemodynamic changes in CA

The evaluation of physiological autoregulation in 8 piglets (weight 1670±191 g) showed that in a wide range of MA BP (around 30 to 100 mmHg), brain NIRS did not changed in the linear regression analysis (r^2^=0.0012), indicating normal autoregulation in each animal after induced hypertension or hypotension. These results were coincident with the blood flow measurements in the superior cava vein, which were not affected by the pressure range used in the experimental model during hypotension or hypertension episodes. During these interventions, neither LVEF (related to myocardial contractility) nor the HR changed. As these determinants of PaCO_2_ show stability during experiments, the modification of brain hemodynamics can be linked to regional changes (Table 1).

**Table 1:** Changes after modification of the systemic arterial pressure (SAP) including responses of superior cava vein blood flow (SCVBF), left ventricular ejection fraction (LVEF), heart rate (HR), and cerebral regional oxygen saturation (rSaO_2_).

	Baseline (previous hypotension)	Hypotensive event	Baseline (previous hypertension)	Hypertensive event	r^2^
SCVBF (m/s)	0.7±0.2	0.7±0.3	0.7±0.2	0.9±0.3	0.00045
LVEF (%)	71±10	60±17	74±9	63±17	0.0052
HR (bpm)	153±24	163±33	162±27	164±37	0.073
Cerebral rSaO_2_ (%)	56±14	60±14	52±5	58±11	0.088
MA BP (mmHg)	58±8	33±12	51±11	85±20	

The relationship between the mean systemic arterial pressure (MA BP) and each variable was analyzed by linear regression, and the resulting r^2^ is reported. Data are reported as means±SD.

### NIRS/Doppler measurements

Interventions were conducted on 6 newborn piglets (weight 1702±191 g). No significant changes on SaO_2_, MA BP, or temperature were registered during the experiment. A statistically significant change in blood pH was observed with hypo- and hyperventilation as well as HR before and after hyperventilation (Table 2).

**Table 2:** Pre- and post-intervention values of assessed variables.

	Baseline	Hyperventilation	Baseline	Hypoventilation
pH	7.46±0.03	7.68±0.08*	7.43±0.03	7.18±0.12*
PaO_2_ (mmHg)	75.8±7.6	80.1±12.7	73.1±9.3	95.4±43.2
Glucose (mg/dL)	122.2±31.6	169.5±83.9	157.2±82.1	144.1±63.1
Hb (g/dL)	8.9±1.7	9.1±1.2	9.0±1.2	9.1±1.7
Hct (%)	27.7±5.2	28.5±3.6	28.1±3.8	28.2±5.3
MA BP (mmHg)	65±13	62±18	74±17	70±15
HR (bpm)	143±30	212±53*	171±48	142±30*
Temperature (°C)	38.5±0.8	38.3±0.5	38.5±0.6	38.7±0.6

Data are reported as means±SD. *P<0.05 compared to baseline (*t*-test). PaO_2_: partial pressure of carbon dioxide; Hb: hemoglobin; Hct: hematocrit; MA BP: mean systemic arterial pressure; HR: heart rate.

**Figure 3:**
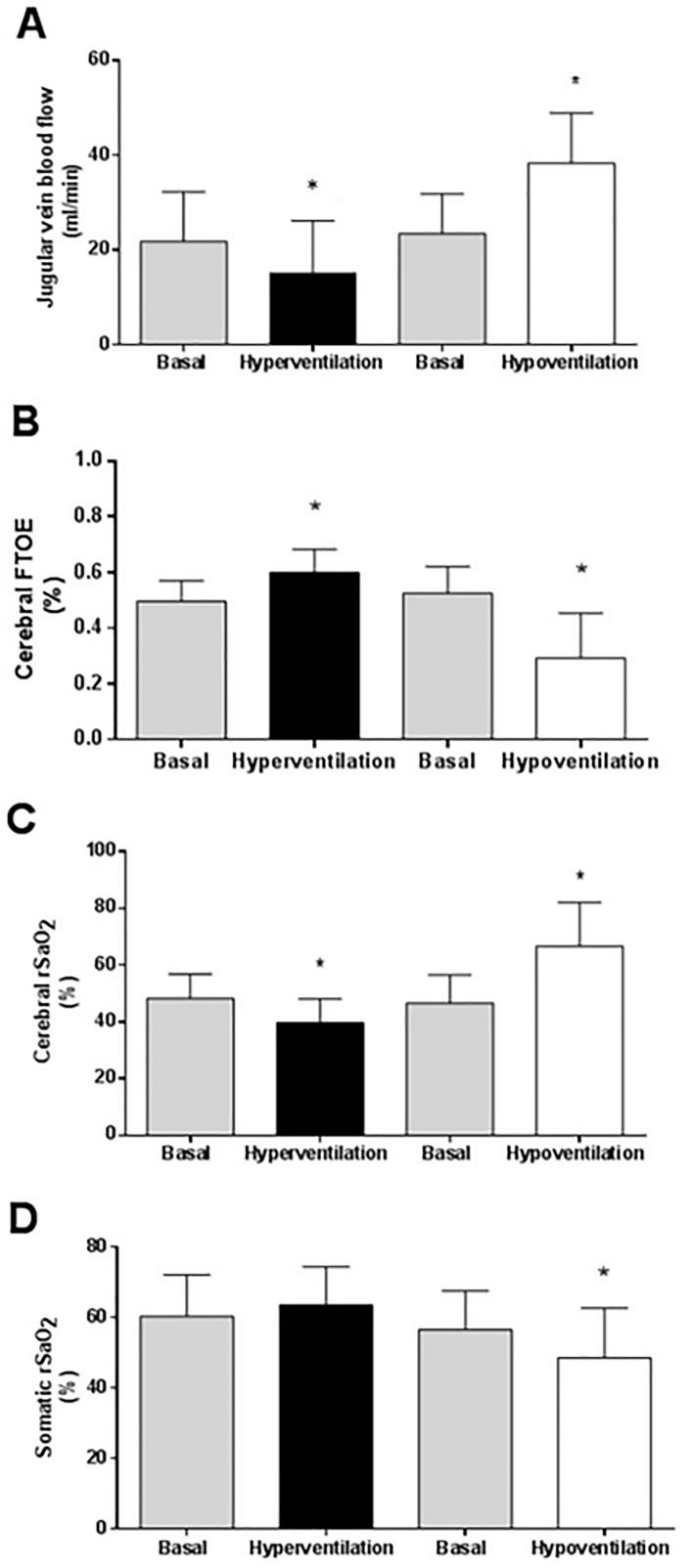
Changes after hyper- and hypoventilation in **A**, jugular vein blood flow; **B**, fractional cerebral tissue oxygen extraction (FTOE); **C**, cerebral oxygen saturation (rSaO_2_); and **D**, somatic rSaO_2._ Data are reported as means±SD. *P<0.05 compared to basal (*t*-test).

A 39% decrease in PaCO_2_ was observed after hyperventilation was induced (baseline 47.6±2.4 *vs* 29.0±4.9 mmHg after the intervention, P<0.01). At the end of the experimentally induced hypercapnia, an 86% increase in baseline PaCO_2_ was observed (baseline 48.5±5.5 *vs* 90.4±25.1 mmHg, P<0.01). The observed changes in PaCO_2_ resulted in a 30% decrease in jugular venous blood flow (JVBF) during the hyperventilation period (baseline 21.8±10.4 mL/min *vs* hyperventilation 15.1±11.0 mL/min, P<0.01) and a 63.5% increase from baseline in the hypoventilation period (baseline 23.4± 8.4 mL/min *vs* hypoventilation 38.3±10.5 mL/min, P<0.01) as shown in Figure 3A. The linear regression analysis showed a direct relationship between changes in PaCO_2_ and changes in JVBF during the interventions of hyper- and hypoventilation (r^2^=0.67, P<0.01) (Figure 3B).

The FTOE also showed significant changes, increasing from 0.49±0.07 at baseline to 0.60±0.08 following hyperventilation and decreasing from 0.49±0.10 to 0.32±0.16 after hypoventilation, due to changes in PaCO_2_ levels. In this analysis, the percentage of variation was +16% at the end of hyperventilation period and -37% after hypoventilation time (Figure 3B).

Cerebral rSaO_2_ during interventions showed significant changes, decreasing from 48±8% at baseline to 39±11% at the end of the intervention in the hypocapnia group and increasing from 46±10% to 67±15% in the hypercapnia group (Figure 3C). The percentage of variation was -17% and +42% for the periods of hyper- and hypoventilation, respectively. The somatic rSaO_2_ showed a significant decrease in hypoventilation (57±11 *vs* 49±15%), without significant modifications in hyperventilation (60±12 *vs* 64±11%), and a percentage of change of +8% and -3.2%, respectively (Figure 3D).

**Figure 4:**
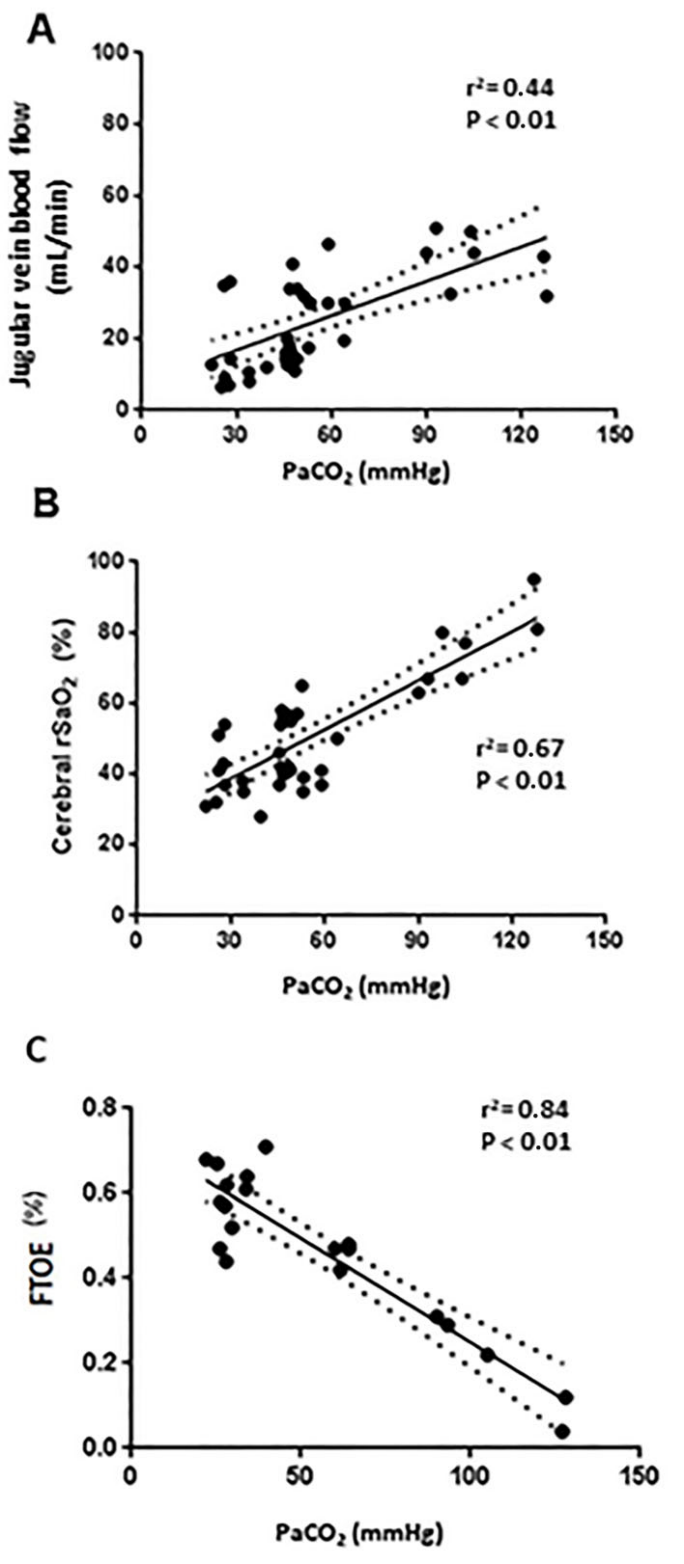
**A,** Linear regression between jugular venous blood flow and partial pressure of carbon dioxide (PaCO_2_) during hyperventilation and hypoventilation maneuvers. **B**, Linear regression between cerebral oxygen saturation (rSaO_2_) and PaCO_2_ during hyperventilation and hypoventilation maneuvers. **C**, Linear regression between cerebral fractional tissue oxygen extraction (FTOE) and PaCO_2_ during hyperventilation and hypoventilation maneuvers.

Linear regression analysis showed a direct relationship between PaCO_2_ and JVBF changes (r^2^=0.44, P<0.01, Figure 4A), with a modification of 7.5 mmHg (1 kPa) for each 1 mL/min of flow change in the IJV, as well as between cerebral rSaO_2_ and PaCO_2_ (r^2^=0.67), with a modification of 24 mmHg for each 1 percent of change in the cerebral rSaO_2_ (Figure 4B). The linear regression analysis showed an inverse relationship between FTOE and PaCO_2_ (r^2^=0.84) as shown in Figure 4C.

**Figure 5:**
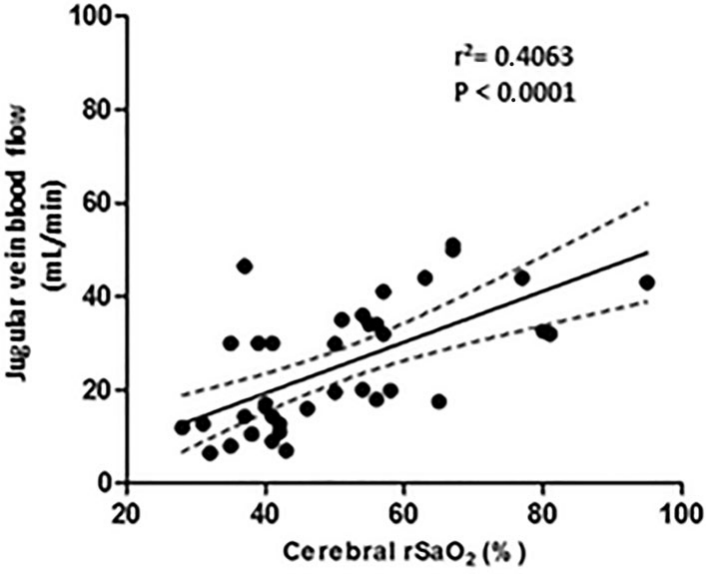
Linear regression between cerebral oxygen saturation (rSaO_2_) and jugular venous blood flow.

Similarly, the linear regression analysis showed a direct relationship between NIRS and JVBF (r^2^=0.40), as shown in Figure 5, with a modification of 6.4% cerebral rSaO_2_ for each 1 mL/min of flow change in the JVBF.

## Discussion

This work showed that monitoring of cerebral rSaO_2_ through NIRS is sensitive to abrupt changes in PaCO_2_ and CBF, specifically to variations in respiratory support, provided no other changes can be attributed to the observed variations.

In this experimental model of newborn piglets, most of the variables that can affect cerebral perfusion were controlled for and PaCO_2_ was modified during the intervention, measuring the changes in rSaO_2_ and JVBF (proxy to CBF).

PaO_2_, blood glucose, SAP, temperature, Hb, and hematocrit did not show significant changes during the interventions.

During hypoventilation (hypercapnia), HR was not substantially modified, but there was a significant increase in this parameter during hyperventilation (hypocapnia). The intervention to produce hypocapnia is based on hyperventilation, and consists of increasing both tidal volume (increase in delta P) and RR, leading to a net increase in minute volume. These changes have an impact on different aspects. The sudden increase of the average airway pressure results in a direct increase of the intrathoracic pressure and, consequently, in a reduction of the systemic venous return. Furthermore, an increase in intrathoracic pressure may increase the resistance to the central venous return, which could be associated with the increase in CBF affecting, in turn, rSaO_2_ (22). As a compensatory mechanism, the stimulation of the sympathetic system determines an increase of the HR (22). Secondly, acute changes in PaCO_2_ impact the autonomic nervous system, resulting in a reduction of the systemic vascular tone. In both cases, the increase in HR serves as a compensatory mechanism (18). Although it is likely that the increase in HR is a transient phenomenon, this cannot be stated in this model as each intervention lasted 15 min.

The changes in PaCO_2_ resulted in significant changes in blood pH, but variations in CBF appear to be determined not by arterial pH but by changes in extravascular pH (by diffusion of CO_2_ into the extravascular space, which changes the pH and consequently modulates muscle tone) (2).

The cerebral blood vessels sensitivity to PaCO_2_ changes was initially demonstrated by Wolff et al. (23). Numerous human studies conducted both in adults and newborns as well as animal models have confirmed the direct relationship between CBF and PaCO_2_ (10,18,24). The observed direct relationship between PaCO_2_ changes and CBF as well as the one observed between cerebral rSaO_2_ and PaCO_2_ is useful as a reference to work with this type of experimental models.

The usefulness of NIRS to evaluate changes in CBF was verified in animal models (24). Accordingly, Booth et al. (17) observed a significant correlation between exhaled CO_2_ and cerebral arterial flow (r^2^=0.51) and between common cerebral artery flow and cerebral rSaO_2_ evaluated with NIRS (r^2^=0.73) in anesthetized newborn piglets subjected to hyper- and hypocapnia.

In human studies conducted on adults who underwent hyperventilation, a 15% decrease in PaCO_2_ and 40% peak cerebral artery flow were observed by TCD, with a significant concomitant 28% decrease in cerebral rSaO_2_ (25,26).

In a retrospective study of 38 preterm newborns (average gestational age of 29.4 weeks), Dix et al. (27) observed a direct relationship between cerebral rSaO_2_ and CO_2_ measured by CO_2_ capnometry at the end of expiration, confirming that CO_2_ fluctuations affect cerebral oxygenation. NIRS ability to identify changes in CBF in ill preterm newborns with persistent ductus arteriosus has also been demonstrated (28). All these changes are consistent with the findings in this experimental model, evidenced by the direct relationship between JVBF and rSaO_2_ in Figure 5.

Although there are several factors that affect cerebral vasoreactivity such as Hb, mechanical ventilation, lower postmenstrual age, male sex, and higher CO_2_ values, it is necessary to move forward in the creation of new knowledge in this scenario in which NIRS can be very useful (8).

Peripheral oxygenation (extracerebral) depends on the balance between oxygen delivery and the metabolic demands of tissues, known as consumption of O_2_. Peripheral oxygen delivery is a complex process that depends on cardiac function, PaO_2_, PaCO_2_, pH, total Hb (and proportion of fetal Hb), blood viscosity, and temperature (29). Animal studies have shown that blood flow at different organs correlate well with somatic rSaO_2_ measurements (30,31).

NIRS has shown to be effective in identifying splanchnic perfusion disturbances in newborns with necrotizing enterocolitis and other abdominal pathologies (32). The brain-splanchnic oxygenation index (rSaO_2_ cerebral/rSaO_2_ splanchnic >0.75) has been established as a predictor of splanchnic ischemia (33). However, there are few studies on extracerebral blood flow showing the effects of hypo- or hypercapnia without hypoxia. Hansen et al. (34) found no significant changes in blood flow of different organs in newborn piglets exposed to changes in PaCO_2_. In our study with piglets in normoxia, a significant decrease in somatic rSaO_2_ was observed during hypercapnia, but not during hypocapnia.

The balance between O_2_ delivery and consumption at the tissue level can be evaluated by FTOE, which correlates well with the O_2_ extraction fraction (6). The O_2_ extraction fraction varies from organ to organ according to its metabolic activity, calculating an overall O_2_ extraction fraction of 0.15-0.33. In other words, between 15% and 33% of the transported O_2_ is consumed (8). In our study, an inverse relationship between PaCO_2_ and cerebral FTOE was observed, with a 37% reduction in O_2_ consumption during hypercapnia and a 16% increase during hypocapnia, confirming previous findings in sheep, piglets, and human newborns (35).

CA under normal circumstances in piglet models has been explored by many authors (34). Our findings confirmed that piglet brain autoregulation was similar to previous studies conducted under similar experimental conditions. In this context, we can assume that oxygen extraction increases when there is a decrease in the rSaO_2_, as the brain tissue compensates the decrease in CBF. As previously demonstrated, this is because rSaO_2_ decreases due to vasoconstriction after hypocapnia, establishing an inverse relationship between FTOE and PaCO_2_ (35).

In this model, variables such as MA BP, temperature, and O_2_ consumption did not present significant modifications, indicating that changes in cerebral rSaO_2_ linked to changes in PaCO_2_ may reflect variations in CBF. The effect on blood flow was particularly evident in extreme values of PaCO_2_ (<35 and >60 mmHg). At these extremes values, the balance between extraction and consumption of O_2_ may be altered if the tissue metabolic rate remains unchanged, which translates into increased FTOE during vasoconstriction and decreased FTOE during vasodilation.

In this sense, consistent findings in animal and human studies have shown an inverse relationship between the increase of PaCO_2_ and consumption of O_2_ (and therefore with O_2_ extraction fraction) (36). A significant increase in PaCO_2_ and a significant reduction in FTOE have also been observed in newborns with increased morbidity and mortality such as necrotizing enterocolitis (37,38).

New technologies such as diffuse optical correlation spectroscopy (DCS) are currently being developed to continuously monitor relative changes in microvascular CBF at the bedside, possibly being more accurate and showing less variability of the measures. DCS could be combined with NIRS to measure CBF and oxygenation continuously, enabling the quantification of cerebral metabolic rate of oxygen (39).

### Limitations of the study

One of the limitations of this study is that our findings cannot be extrapolated to human clinical practice. When managing unstable patients with complete monitoring, there are multiple variables to take into account, including fluctuations shown on NIRS recordings related to PaCO_2_ or CBF changes.

Another limitation of the study is that the most frequently used method for quantifying CBF, the TCD ultrasound, was not available for this experimental study. However, variations in CBF can be indirectly estimated by IJV Doppler ultrasound, taking into consideration that a fraction of such flow returns via the vertebral veins.

### Conclusions

Sudden changes in PaCO_2_ secondary to mechanical ventilation determine abrupt changes in cerebral perfusion, which can be detected by NIRS. This is a useful method of continuous bedside monitoring of patients that is more accessible and cost-effective than TCD.

Therefore, NIRS is a tool that could influence decision-making in a timely manner in the prevention of encephalic alterations associated to hemodynamic instability and consequent changes in CBF.
